# Adiposity, adult weight change and breast cancer risk in postmenopausal Japanese women: the Miyagi Cohort Study

**DOI:** 10.1038/sj.bjc.6605885

**Published:** 2010-09-14

**Authors:** M Kawai, Y Minami, S Kuriyama, M Kakizaki, Y Kakugawa, Y Nishino, T Ishida, A Fukao, I Tsuji, N Ohuchi

**Affiliations:** 1 Department of Surgical Oncology, Tohoku University Graduate School of Medicine, 1-1 Seiryo-machi, Aoba-ku, Sendai, Miyagi 980-8574, Japan; 2 Division of Community Health, Tohoku University Graduate School of Medicine, 2-1 Seiryo-machi, Aoba-ku, Sendai, Miyagi 980-8575, Japan; 3 Division of Epidemiology, Department of Public Health and Forensic Medicine, Tohoku University Graduate School of Medicine, 2-1 Seiryo-machi, Aoba-ku, Sendai, Miyagi 980-8574, Japan; 4 Division of Surgery, Miyagi Cancer Center, 47-1 Nodayama, Medeshima-Shiode, Natori, Miyagi 981-1293, Japan; 5 Division of Epidemiology, Miyagi Cancer Center Research Institute, 47-1 Nodayama, Medeshima-Shiode, Natori, Miyagi 981-1293, Japan; 6 Department of Public Health, Yamagata University School of Medicine, 2-2-2 Iida-nishi, Yamagata 990-9585, Japan

**Keywords:** breast cancer, cohort study, body weight, body mass index, incidence

## Abstract

**Background:**

The role of adult weight change in breast cancer (BC) risk is unclear in Japanese women.

**Methods:**

A total of 10 106 postmenopausal women aged 40–64 years (the Miyagi Cohort) were followed from 1990 to 2003, and 108 BC cases were identified. Hazard ratios (HRs) were estimated according to body mass index (BMI) at the current age and at the of age 20 years, and weight change since age 20 years.

**Results:**

Higher current BMI was associated with an increased risk of BC (*P* for trend=0.02), whereas higher BMI at the age 20 years was inversely associated with this risk (*P* for trend=0.002). There was a significant association between weight change since age 20 years and BC risk (*P* for trend=0.0086). Compared with stable weight, HR was 0.35 for weight loss of 5 kg or more (*P* for weight loss trend=0.04) and 1.55 for weight gain of 12 kg or more (*P* for weight gain trend=0.05).

**Conclusion:**

Adiposity at younger and current age has differential effects on BC risk among postmenopausal women; weight gain in adulthood being associated with an increased, and weight loss with a decreased risk.

The incidence of breast cancer (BC) shows variations among countries and although Japan has a lower risk of BC than Western countries, its age-standardised incidence is the highest among female cancers, and it is increasing (Matsuda *et al*, 2008). The increase of BC incidence may be attributed to a change in the proportion of women in the population who have reproductive and anthropometric risk factors (Minami *et al*, 2004). Among such risk factors, the associations between adiposity and BC risk have been extensively investigated, mainly in the Western countries (Lahmann *et al*, 2004; Morimoto *et al*, 2002; Reeves *et al*, 2007). In relation to adiposity, weight gain has also been associated with an increased risk of postmenopausal BC in several prospective studies (Ahn *et al*, 2007; Barnes-Josiah *et al*, 1995; Eliassen *et al*, 2006; Feigelson *et al*, 2004; Lahmann *et al*, 2005). In Japan, however, few prospective studies have evaluated the association with adiposity (Iwasaki *et al*, 2007; Kuriyama *et al*, 2005). Also, data are sparse regarding the effect of body weight change (Hirose *et al*, 1999; Kyogoku *et al*, 1990).

We therefore conducted a population-based cohort study, in which we evaluated the association of adiposity in different periods, that is, at current age and at age 20 years, with BC risk and examined the change in risk resulting from body weight gain and loss since the age of 20 years among postmenopausal Japanese women.

## Materials and methods

Our analysis used the Miyagi Cohort Study, whose design has been described in detail elsewhere (Fukao *et al*, 1995; Kawai *et al*, 2010). Briefly, 25 279 men and 26 642 women aged 40–64 years living in 14 municipalities, selected randomly from among the 62 municipalities in Miyagi Prefecture, Northeastern Japan, were entered into a cohort on 1 June 1990. A self-administered questionnaire on various health aspects was delivered to these subjects between June and August 1990. Usable questionnaires were returned by 22 836 men (90.3%) and 24 769 women (93.0%). After excluding men, women with a history of cancer (*n*=705), who were premenopausal (*n*=9131), with undefined menopausal status (*n*=642) and for whom data on menopausal status were missing (*n*=2927), 11 364 postmenopausal women remained (Kawai *et al*, 2010). After further excluding women with missing data or extreme values for current height or current weight or weight at age 20 years (*n*=1258), 10 106 postmenopausal women contributed to this study. The study protocol was approved by the institutional review board of Tohoku University School of Medicine. We considered the return of self-administered questionnaires signed by the subjects to imply their consent to participate in the study.

The questionnaire covered personal history including current height (centimeters) and weight (kilograms) and weight at age 20 years and details of general lifestyle including menstrual and reproductive histories. The self-reported current height and weight data were highly correlated with measured data (correlation coefficient: 0.82 for height and 0.97 for weight) in a subsample of postmenopausal women (*n*=2921), although we were unable to validate the data for weight at age 20 years.

As a measure of adiposity, body mass index (BMI) was used. The BMI at the current age and at age 20 years, calculated as weight divided by the square of current height (kg m^–2^), respectively. To analyze BC risk for adiposity in the different periods, the study women were categorised using quartile points of BMI at age 20 years, respectively: <20.5, ⩾20.5–<22.0, ⩾22.0–<23.8 and ⩾23.8. Subjects with a current BMI of 23.8 and higher were further divided into two groups on the basis of median value in the range between 23.8 and the largest current BMI, as the BMI at the current age was skewed towards a higher value than at age 20 years. Finally, women were categorised as follows: current BMI <20.5, ⩾20.5–<22.0, ⩾22.0–<23.8, ⩾23.8–<25.9 and ⩾25.9; BMI at age 20 <20.5, ⩾20.5–<22.0, ⩾22.0–<23.8 and ⩾23.8. Weight change from age 20 years to the current age was calculated as the difference between current weight and weight at age 20. Subjects were also categorised into seven groups as follows: weight loss of ⩽–5 and >–5 to ⩽–2, stable weight of >–2 to <+2, and weight gain of ⩾+2 to <+5, ⩾+5 to <+8, ⩾+8 to <+12 and ⩾+12. The categorisation of weight loss was based on the median value, and that of weight gain was determined using quintile values.

Women were followed from the start of the study (1 June 1990) until 31 December 2003. The end point of our analysis was BC defined as the topography codes C50.0–C50.9 according to the International Classification of Disease for Oncology, Second Edition, and confirmed by the Miyagi Prefecture Cancer Registry, one of the oldest and most accurate population-based in Japan (Curado *et al*, 2007). In this registry, the percentage registered by death certificates only for BC was 2.5% during 1991–2003. A follow-up committee was also established, consisting of the Miyagi Cancer Society, the Divisions of Community Health of all 14 municipalities, the Department of Health and Welfare, Miyagi Prefectural Government, and the Division of Epidemiology, Tohoku University School of Medicine. The committee periodically reviewed the residential registration record of each municipality. During the study period, 491 women (4.9%) were lost to follow-up because of emigration.

### Statistical analysis

The person-years of follow-up were counted for each of the subjects from the start of the study (1 June 1990) until the date of diagnosis of BC, the date of emigration from the study area, the date of death, or the end of follow-up (31 December 2003), whichever occurred first. The mean follow-up period was 12.8 years. The Cox proportional–hazard regression model was used to estimate BC hazard ratios (HRs) and 95% confidence intervals (CIs) according to category of exposure variable, that is, BMI at the current age, BMI at age 20 years and weight change from age 20 years to the current age, and to adjust for confounding variables (Cox, 1972). Linear trends, which were tested using the Cox model by treating each exposure category as a continuous variable, were regarded as significant if *P*-values were <0.05. We considered the following variables as potential confounders: age, education level, cigarette smoking, alcohol drinking, and time spent walking, which are known or suspected risk factors for BC. Menstrual and reproductive factors, exogenous female hormone use, and history of BC in the mother or sisters, some of which had been established as risk factors in our previous study (Kawai *et al*, 2010), were also considered to be adjusted for each other. In the analysis of weight change, height and weight at age 20 years were further adjusted for (Eliassen *et al*, 2006). Missing values for confounders were treated as an additional variable category, and were included in the model. To evaluate any independent effect of BMI during the different periods, analysis adjusting for both BMIs each other was also conducted. All statistical analyses were performed using the SAS software package (version 9.1; SAS Institute, Cary, NC ,USA).

## Results

The characteristics of the study subjects are presented in Tables 1 and 2. The subjects with a higher current BMI were less likely to smoke, whereas the subjects with a higher BMI at age 20 years tended to be older and to have a shorter period of education (Table 1). A total of 64.8% of the subjects had gained more than 2 kg since age 20 years (Table 2). The subjects who lost weight were heavier at age 20 years.

During 129 891 person-years of follow-up, 108 BC cases were documented. Table 3 shows the HRs and 95% CIs according to current BMI and BMI at age 20 years. After adjustment for confounding variables, current BMI was marginally associated with an increased BC risk (*P* for trend in multivariate-adjusted model 1=0.07). The BMI at age 20 years was inversely associated risk (*P* for trend in multivariate-adjusted model 1=0.01). Postmenopausal women with a BMI of ⩾23.8 at age 20 years showed half the risk (multivariate-adjusted HR=0.44, 95% CI: 0.24–0.81) of women with a BMI of <20.5. Multivariate analysis adjusting for both BMIs each other demonstrated a stronger inverse association for BMI at age 20 years (*P* for trend in multivariate-adjusted model 2=0.002). The association of current BMI with risk was statistically significant (*P* for trend=0.02).

Weight change since the age of age 20 years was significantly associated with the risk (multivariate-adjusted *P* for trend=0.0086) (Table 4). Compared with women whose weight had been stable (lost or gained <2.0 kg), those who lost 5 kg or more were at a lower risk (multivariate-adjusted HR 0.35, 95% CI: 0.11–1.10). Women with a weight gain of 12 kg or more appeared to have a higher risk (HR 1.55, 95% CI: 0.70–3.45). According to weight loss and gain, weight loss was associated with a decreased risk (*P* for weight loss trend=0.04), and weight gain with an increased risk (*P* for weight gain trend =0.05). Although the data are not shown in the table, stratified analysis by the BMI at age 20 years revealed a clearer inverse association with weight loss among women who were heavier at age 20 years (BMI at age 20 years ⩾23.8; *P* for weight loss trend=0.01).

## Discussion

In this population-based cohort study, we found associations between adulthood adiposity and weight change and BC risk among postmenopausal women. Risk differed for BMI between that at current age and that at age 20 years. Weight change from age 20 to current age was significantly associated with risk. These results provide some insight into the significance of adiposity and weight change in terms of BC risk in postmenopausal Japanese women.

This study found a positive association of current BMI with postmenopausal BC risk consistent with previous prospective studies (Iwasaki *et al*, 2007; Kuriyama *et al*, 2005; Lahmann *et al*, 2004; Morimoto *et al*, 2002; Reeves *et al*, 2007), and the fact that postmenopausal obese women have more oestrogens than lean women (Potischman *et al*, 1996), has a central role in BC aetiology. After menopause, oestrogen is synthesised mainly by aromatase in adipose tissue (Bulun *et al*, 2005). Another mechanism is that obese women may be in a state of hyperinsulinemia, insulin being a growth factor for BC cells. Insulin-like growth factor I may also affect risk among heavier women (Muti *et al*, 2002). On the other hand, a higher BMI at age 20 years was significantly associated with a decreased postmenopausal risk. This inverse association, which has also been observed in the Western countries (Ahn *et al*, 2007; Morimoto *et al*, 2002; Sellers *et al*, 1992; van den Brandt *et al*, 1997), was independent of the effect of current BMI. The Nurses’ Health Study recently reported the independent protective effect of body fatness at young age using a pictogram (Baer *et al*, 2010). Although the mechanisms explaining this inverse association are poorly understood, lower serum oestradiol and progesterone levels and anovulation among young obese women may reduce BC risk after menopause (Potischman *et al*, 1996).

There was a significant association between weight change since age 20 and postmenopausal risk. Weight gain was associated with an increased risk, and weight loss with a decreased risk. For postmenopausal women who were heavier at age 20 years, a clearer inverse association with weight loss was observed. These results might have been expected from the different associations of risk with BMI at the current age and at age 20 years, as mentioned above. To our knowledge, this is the first prospective cohort study to have evaluated the association between weight change and BC risk among Japanese women. The positive effect of weight gain has been observed in nearly all prospective studies from Western countries (Ahn *et al*, 2007; Barnes-Josiah *et al*, 1995; Eliassen *et al*, 2006; Feigelson *et al*, 2004; Lahmann *et al*, 2005). On the other hand, the relationship of weight loss to risk has not been fully investigated (Eliassen *et al*, 2006; Harvie *et al*, 2005; Hirose *et al*, 1999; Kyogoku *et al*, 1990; Lahmann *et al*, 2005). Although most of the studies have observed a null or non-significant association, a few have demonstrated a significantly decreased postmenopausal risk associated with adult weight loss (Eliassen *et al*, 2006). Our results provide additional evidence for the association with adult weight change, especially weight loss.

Strengths of our study included its prospective design and the high quality of the follow-up. Although this cohort was relatively small scale, participants were recruited from the general population, and BC cases were identified from the population-based cancer registry. Furthermore, the rate of loss to follow-up was low, so selection and information bias were avoided. Limitations included, first, the fact that weights at age 20 years and at current age, and height, were self-reported. The correlations between measured and self-reported current weight and height were high. On the other hand, there were no data for measured weight at age 20years. The self-recalled weight at age 20 years may have been lower or higher than the real weight, thus causing a non-differential misclassification bias. However, it is unlikely that this bias would have seriously distorted the results (Rothman and Greenland, 1998). Second, our results may have been contaminated subclinical effects of BC by cases occurring soon after recruitment. We, therefore, analyzed the data after omitting cases that occurred within 2 years of recruitment, but this yielded almost the same results (data not shown).

This study has found that adiposity at younger and current age has differential effects on BC risk, and that weight change during adulthood is associated with the postmenopausal risk among Japanese women; weight gain was associated with an increased risk, and weight loss with a decreased risk. As body weight is a modifiable lifestyle factor, weight control throughout life appears to be useful in BC.

## Figures and Tables

**Table 1:** Characteristics of study population according to body mass index (BMI)

	**<20.5**	**⩽20.5 <22.0**	**⩽22.0 <23.8**	**⩽23.8 <25.9**	**⩽25.9**
*Current BMI*					
Number of subjects	1209	1497	2335	2516	2549
Age (mean, years)	57.2±4.5	57.0±4.4	57.1±4.3	57.2±4.3	57.5±4.2
Occupation (no occupation/housewife, %)	15.4	15.5	17.3	16.6	17.1
Educational level (college/university or higher, %)	12.4	12.8	12.5	11.7	10.3
Alcohol drinking (drinkers, %)	19.5	19.0	18.6	19.1	18.8
Smoking (smokers, %)	10.3	7.6	5.9	5.7	5.8
Walking status (<1 h per day, %)	44.7	46.6	45.1	46.1	49.2
Family history of breast cancer in mother or sisters (%)	1.8	1.7	2.3	2.0	2.0
Age at menarche (mean, years)	15.4±2.1	15.3±1.9	15.3±2.0	15.2±2.0	15.2±2.1
Age at menarche (16 years ⩽, %)	37.2	35.9	36.2	34.7	32.4
Age at natural menopause (mean, years)	49.1±3.8	49.5±3.6	49.4±3.4	49.4±3.7	49.7±3.7
Parity (nulliparous, %)	3.7	2.7	1.8	2.3	2.2
Parity number among parous women (mean)	2.6±1.0	2.6±1.0	2.7±1.0	2.7±1.0	2.8±1.1
Exogenous female hormone use (users, %)	11.7	10.6	9.9	10.8	10.0
Height (mean, cm)	152.4±6.7	151.7±5.2	151.5±5.0	151.5±4.9	150.7±5.3
					
	**<20.5**	**⩽20.5 <22.0**	**⩽22.0 <23.8**	**⩽23.8**	
*BMI at age 20 years*					
Number of subjects	2577	2460	2594	2475	
Age (mean, years)	56.9±4.4	57.1±4.3	57.3±4.3	57.6±4.2	
Occupation (no occupation/housewife, %)	19.0	17.1	16.3	14.0	
Educational level (college/university or higher, %)	14.0	12.7	10.8	9.5	
Alcohol drinking (drinkers, %)	20.0	17.5	18.6	19.6	
Smoking (smokers, %)	7.3	6.0	6.1	6.8	
Walking status (<1 h per day, %)	48.7	45.6	47.0	44.7	
Family history of breast cancer in mother or sisters (%)	2.3	1.5	2.1	2.0	
Age at menarche (mean, years)	15.4±2.0	15.2±1.9	15.3±2.0	15.3±2.1	
Age at menarche (16 years ⩽, %)	36.7	33.3	35.0	34.7	
Age at natural menopause (mean, years)	49.3±3.7	49.4±3.5	49.5±3.7	49.6±3.6	
Parity (nulliparous, %)	3.7	2.0	2.0	1.8	
Parity number among parous women (mean)	2.6±1.0	2.7±1.0	2.7±1.1	2.8±1.1	
Exogenous female hormone use (users, %)	11.6	9.9	10.2	10.1	
Height (mean, cm)	152.9±5.8	151.6±4.7	151.0±5.0	150.2±5.4	

**Table 2:** Characteristics of study population according to weight change from age 20 to the current age

**Characteristics**	**Weight loss^a^**	**Stable weight^b^**	**Weight gain^c^**
Number of subjects	2801	758	6547
Age (mean, years)	57.4±4.3	57.1±4.4	57.2±4.3
Occupation (no occupation/housewife, %)	14.6	16.1	17.5
Education level (college/university or higher, %)	10.6	13.6	12.0
Alcohol drinking (drinkers, %)	19.4	18.2	18.9
Smoking (smokers, %)	8.0	6.3	6.0
Walking status (<1 h per day, %)	43.5	44.7	48.1
Family history of breast cancer in mother or sisters (%)	1.6	3.2	2.0
Age at menarche (mean, years)	15.4±2.0	15.1±1.9	15.2±2.0
Age at menarche (16 years ⩽, %)	37.9	30.0	34.3
Age at natural menopause (mean, years)	49.5±3.6	49.3±3.4	49.5±3.6
Parity (nulliparous, %)	2.2	3.0	2.4
Parity number among parous women (mean)	2.7±1.0	2.7±1.0	2.7±1.0
Exogenous female hormone use (users, %)	10.2	12.4	10.3
Height (mean, cm)	150.8±5.5	151.3±5.2	151.7±5.3
Weight at 20 years (mean, kg)	54.7±6.2	51.3±6.1	49.0±5.4
Current body mass index (mean)	21.6±2.3	22.4±2.4	25.2±2.9

Weight change was evaluated for subjects with complete data for height.

a Weight loss ⩾2 kg.

b Weight gain or loss <2 kg.

c Weight gain ⩾2 kg.

**Table 3:** Hazard ratio (HR) and 95% confidence interval (CI) of breast cancer according to current body mass index (BMI) and BMI at age 20 years

			**Age-adjusted model**		**Multivariate-adjusted model 1^a^**		**Multivariate-adjusted model 2**	
	**Person-years**	**Cases**	**HR**	**95% CI**	**HR**	**95% CI**	**HR**	**95% CI**
*Current BMI*								
<20.5	15 327	8	1.00	(Reference)	1.00	(Reference)	1.00	(Reference)^b^
20.5⩽ <22.0	19 121	15	1.50	0.64–3.54	1.51	0.64–3.56	1.63	0.69–3.86
22.0⩽ <23.8	29 835	24	1.54	0.69–3.43	1.55	0.70–3.46	1.74	0.78–3.90
23.8⩽ <25.9	32 575	27	1.59	0.72–3.49	1.64	0.74–3.61	1.86	0.84–4.12
25.9⩽	33 033	34	1.97	0.91–4.25	2.04	0.94–4.41	2.54	1.16–5.55
*P* for trend				0.09		0.07		0.02
								
*BMI at age 20 years*								
<20.5	32 880	37	1.00	(Reference)	1.00	(Reference)	1.00	(Reference)^c^
20.5⩽ <22.0	31 555	29	0.82	0.50–1.33	0.88	0.54–1.44	0.83	0.51–1.36
22.0⩽ <23.8	33 460	28	0.74	0.45–1.21	0.80	0.49–1.31	0.72	0.44–1.19
23.8⩽	31 996	14	0.39	0.21–0.72	0.44	0.24–0.81	0.38	0.20–0.70
*P* for trend				0.003		0.01		0.002

a Adjusted for age (continuous variable), alcohol drinking (ever, never), smoking (ever, never), occupation (permanent, no occupation/housewife), walking (<1 h per day, longer than 1 h per day), education level (junior high school or less, high school, college/university or higher), age at menarche (⩽13, 14, 15, 16<), age at menopause (⩽47, 48⩽ ⩽50, 51⩽ ⩽53, 54⩽), parity number (0, 1, 2, 3, 4, 5⩽), family history of breast cancer (present, absent) and history of exogenous female hormone use (ever, never).

b Additionally adjusted for BMI at age 20 years (<20.5, 20.5⩽ <22.0, 22.0⩽ <23.8, 23.8⩽).

c Additionally adjusted for current BMI (<20.5, 20.5⩽ <22.0, 22.0⩽ <23.8, 23.8⩽ <25.9, 25.9⩽).

**Table 4:** Hazard ratio (HR) and 95% confidence interval (CI) of breast cancer according to weight change from age 20 years to the current age

			**Age-adjusted model**		**Multivariate-adjusted model^a^**	
**Weight change (kg)**	**Person-years**	**Cases**	**HR**	**95% CI**	**HR**	**95% CI**
⩽−5	19760	5	0.31	0.10–0.94	0.35	0.11–1.10
−5< ⩽−2	16128	13	0.98	0.41–2.36	1.05	0.43–2.55
−2< <+2	9714	8	1.00	(Reference)	1.00	(Reference)
+2⩽ <+5	18765	11	0.71	0.29–1.77	0.70	0.28–1.75
+5⩽ <+8	22857	21	1.12	0.49–2.52	1.09	0.48–2.47
+8⩽ <+12	20100	19	1.15	0.50–2.62	1.10	0.48–2.53
+12⩽	22567	31	1.67	0.77–3.63	1.55	0.70–3.45
*P* for trend				0.0002		0.0086
*P* for weight loss trend				0.03		0.04
*P* for weight gain trend				0.02		0.05

a Adjusted for age (continuous variable), height (<149, 149⩽ <152, 152⩽ <156, 156⩽), body weight at age 20 (continuous variable), alcohol drinking (ever, never), smoking (ever, never), occupation (permanent, no occupation/housewife), walking (<1 h per day, longer than 1 h per day), education level (junior high school or less, high school, college/university or higher), age at menarche (⩽13, 14, 15, 16<), age at menopause (⩽47, 48⩽ ⩽50, 51⩽ ⩽53, 54⩽), parity number (0, 1, 2, 3, 4, 5⩽), family history of breast cancer (present, absent) and history of exogenous female hormone use (ever, never).
